# The extragonadal biology of gonadotropin hormones: should we rename these?

**DOI:** 10.1530/EC-26-0232

**Published:** 2026-08-24

**Authors:** Se-Min Kim, Funda Korkmaz, Sorang Kang, Fazilet Sen, Steven L Sims, Judit Gimenez-Roig, Uliana Cheliadinova, Darya Vasilyeva, Orly Barak, Susan Hutchison, Anne Macdonald, Rauf Latif, Ki Goosens, Tal Frolinger, Anusha Pallapati, Satish Rojekar, Vitaly Ryu, Daria Lizneva, Tony Yuen, Mone Zaidi

**Affiliations:** ^1^ Institute of Translational Medicine and Pharmacology, Icahn School of Medicine at Mount Sinai, New York, New York, USA; ^2^ Rutgers Health, Jersey City Medical Center, Jersey City, New Jersey, USA; ^3^ Department of Medicine, Englewood Hospital and Medical Center, Englewood, New Jersey, USA; ^4^ Turkish Medicines and Medical Devices Agency, Ankara, Türkiye

**Keywords:** pituitary, extragonadal effect, gonadotropin, FSH, LH

## Abstract

Over recent years, gonadotropins and their receptors have been shown to exert non-traditional actions that bypass the classical hypothalamic–pituitary–gonadal axis. Findings of the expression of receptors for follicle-stimulating hormone (FSH) and luteinizing hormone (LH) in skeletal, fat, and immune cells and in neurons suggest that their functions are much broader than their classical roles. FSH, once believed solely to regulate procreation–gonadal development and maturation at puberty and gamete production during the fertile phase, has been found to regulate body composition, bone metabolism, and cognition, providing the underpinnings of complex integrative physiology, and, in turn, opening potential new therapeutic opportunities. Pre-clinical evidence from genetic and pharmacologic interventions in rodent models and human data from population-based observations, genetic studies, and a small number of interventional studies together support the independent skeletal, adipogenic, and cognitive actions of gonadotropins. Here, we review our current understanding of direct actions of FSH and LH on bone, adipose tissue, and brain and focus specifically on the detrimental health burden during and after the menopause: osteoporosis, obesity, and dementia.

## Structure and regulation of gonadotropins

The heterodimeric glycoprotein hormones, follicle-stimulating hormone (FSH), luteinizing hormone (LH), and human chorionic gonadotrophin (hCG), which share structural homology, belong to an evolutionarily conserved glycoprotein hormone family. These are non-covalently linked heterodimeric glycoproteins that consist of a common α subunit and a specific β subunit; the latter generates unique biologic activity.

The *FSHB* and *LHB* genes are located on chromosomes 11 and 19, respectively (1, 2). The *FSHB* gene encodes 18 signal peptides followed by 111-amino-acid-long mature peptides with two glycosylation sites, Asn 7 and Asn 24, which play critical roles in hormone signaling (3, 4). Acidic isoforms with high numbers of sialic acid residues have less specific activity with slower hepatic clearance. Estrogen regulates the mix of isoforms, with acidic isoforms becoming dominant after the menopause (3). The *LHB* gene encodes a 24-amino-acid-signal-long peptide followed by 121-amino-acid-long mature protein with one glycosylation site (Asn 30) (2, 5).

The secretion of FSH and LH is regulated by coordinated action of the hypothalamus, pituitary gland, and peripheral gonads. The pulsatile rather than continuous release of GnRH into the hypophyseal portal circulation results in episodic FSH and LH release (6). The activation of GnRH receptor (GnRHR), a rhodopsin G protein-coupled receptor, induces gonadotropin secretion through extracellular Ca^2+^ influx (7). The differential regulation of FSH and LH secretion is mediated by variations in GnRH pulse amplitude and frequency.

Intra-pituitary paracrine factors, such as activin, inhibin, and follistatin, play key roles in the differential regulation of FSH and LH secretion. Although inhibins are produced by the gonads, activins are synthesized within the pituitary and act locally as autocrine and paracrine regulators. Inhibin A is high during the late follicular and luteal phase, while inhibin B rises during the late luteal and early follicular phases of the menstrual cycle. In males, inhibin B is produced by the testes in response to FSH and provides negative feedback on FSH secretion (8, 9). Follistatin, an activin-binding protein, inhibits activin action by interfering with activin binding to its receptor (9).

Gonadal steroid hormones such as estrogens, progesterones, and androgens act directly at the level of the gonadotroph and indirectly *via* effects at the hypothalamus that modulate GnRH secretion. Estrogens can exert dual-feedback effects on gonadotropin secretion, directly suppressing the expression of *FSHB* and *LHB*, as well as generating negative feedback on hypothalamus mediated, in large part, through effects on kisspeptin neurons (8, 10). Progesterone suppresses the GnRH pulse, thereby affecting pituitary gonadotropin secretion. Testosterone serves as the primary negative feedback on gonadotropin secretion in males. Similar to estrogen, this inhibition seems to be mediated by kisspeptin neurons in the hypothalamus (8, 10).

## Functions of gonadotropins in procreation

FSH and LH play an essential role in reproduction. They act on the ovaries and testes to direct gametogenesis and steroid hormone synthesis. In 1929, Zondek discovered that the pituitary gland secretes two hormones that stimulated gonads, prolan A and prolan B, which *hitherto* were termed FSH and LH, respectively (11). FSH was first isolated from the sheep pituitary, and injections of FSH were shown to enlarge ovarian follicles in hypophysectomized rats. In contrast, LH had no effect when given alone but luteinized the follicles after FSH stimulation (12).

In females, FSH acts on FSH receptors (FSHRs) on granulosa cells to enhance follicular cell growth and stimulate estradiol synthesis. During the follicular phase, it regulates the production of inhibin and induces LH receptor (LHCGR) expression in granulosa cells of large preovulatory follicles. While promoting the selection and development of the dominant follicle, FSH simultaneously recruits and enhances the next cohort of follicles for subsequent cycles (13). LH, acting on the LH–human chorionic gonadotropin receptor (LHCGR) in ovarian theca cells, is a major regulator of ovarian steroid synthesis. It stimulates the production of estrogen and androgen precursors in theca cells, which are then aromatized into estrogen in response to FSH. LH also stimulates the expression of progesterone receptors in the granulosa cells of the dominant follicle, which promotes luteinization (2). In males, LH binds to LHCHRs on Leydig cells and stimulates testosterone production. FSH, acting in concert with the intratesticular testosterone, is essential for spermatogenesis. In Sertoli cells, FSH synthesizes inhibin, androgen-binding protein, and androgen receptors, promoting spermatogenesis (14).

## Extragonadal expression of FSH receptors

Apart from their expression in granulosa cells of the ovary and Sertoli cells of the testis, it has become clear that FSHRs are expressed more ubiquitously, in bone and fat cells, as well as in neurons in multiple brain regions (15, 16, 17, 18) (Table 1). The FSHR has 10 exons with the first 9 exons encoding the extracellular domain and exon 10 encoding the transmembrane domain (19, 20, 21). A splice variant lacking exon 9, expressed in osteoclasts and adipocytes, interacts with an inhibitory Gi protein rather than a stimulatory Gs (19, 20, 21). This splice variant is also found in human monocytes, but at significantly lower levels compared with ovarian cells (22).

**Table 1:** Expression of FSHR in Bone, Fat, and Brain.

Tissue/cells	Species	Method	Result
Bone (17, 42)	Mice	*In vivo* NIR-II fluorophore imaging using FSH-CH1055	(+)
Osteoclasts (RAWC3 and RAW 264.7 cells) (41)	Mice	RT-PCR	(+)
Bone, osteoblasts, and osteoclasts (47)	Mice	RT-PCR (exons 7–10)	(−)
Bone, osteoclasts, and RAW 264.7 cells (48)	Mice	RT-PCR (N/A), WB	(−)
Mesenchymal stem cells and osteoclasts (41)	Human	RT-PCR, WB, FACS, immunolabeling using human anti-FSHR (Zymed, USA)	(+)
Monocytes, macrophages, and osteoclasts (19)	Human	RT-PCR (exons 2–3 and exons 8–10), WB	(+)
Osteoclasts and CD14+ cells (22)	Human	RT-PCR and Sanger sequencing (full-length and isoform missing exon 9)	(+)
Osteoblasts (41)	Human	RT-PCR	(−)
Osteoblasts (19)	Human	WB	(−)
Adipocytes (MSC and 3T3.L1 cells) (16)	Mice	Sanger sequencing, cAMP assay (with FSH)	(+)
sWAT, vWAT, and BAT (16)	Mice	IHC (Lifespan, USA, Cat. #LS-A4004)	(+)
sWAT and vWAT (58)	Boar	RT-PCR	(+)
sWAT (57)	Chicken	RT-PCR, IHC (Huijia Biological Technology, China)	(+)
sWAT (54)	Human, mice	IHC, RT-PCR	(+)
Adipocytes, vWAT, and sWAT	Human	WB, IHC (FSHR323 ab), and RNAscope	(−)
Brain (cerebral cortex, hippocampus, forebrain, etc.) (77)	Mice	RNAscope	(+)
Brain cortex and hippocampus, rat neuron (15)	Mice and rat	RT-PCR, WB (Thermo Fisher, USA, PA5-50963), IHC, and RNAscope	(+)
Hypothalamus (58)	Boar	RT-PCR	(+)
Brain cortex and neuroblastoma (SH-SY5Y) (15)	Human	RT-PCR, viewRNA analysis	(+)

WB, western blot; vWAT, visceral white adipose tissue; sWAT, subcutaneous white adipose tissue; BAT, brown adipose tissue; MSC, mesenchymal stem cell; NIR, near-infrared.

In the human female reproductive tract and placenta, FSHRs are expressed in vascular endothelial cells, endometrial glands, cervical glands and stroma, stromal cells, and muscle fibers, as well as in interstitial macrophages from testis (23, 24). FSHR expression can be high in endothelial cells residing within various cancer tissues, including breast, colon, pancreas, and kidney cancers (25).

Our data using RNAscope showed *Fshr* transcript expression in multiple brain regions in mice and humans, particularly in the cerebral cortex and the granular layer and pyramidal tract of the hippocampal dentate gyrus (15), suggesting, therefore, a direct role of FSH–FSHR interactions in cognition.

## High FSH causes bone loss

An independent skeletal effect of FSH in people can be inferred by observations in specific groups, such as women during the perimenopausal transition, patients with Turner syndrome, or even elderly cohorts. Notably, rapid and substantial bone loss occurs around 2–3 years prior to the final menstrual period, coinciding with relatively normal serum estrogen levels and escalating FSH levels (26, 27). This rapid bone loss cannot conceivably be explained by low estrogen, which led us to evaluate FSH as a possible culprit.

Furthermore, a set of evolving epidemiologic studies has suggested that FSH is a strong predictor of bone mineral density (BMD) loss. Notably, the Study of Women’s Health Across the Nation (SWAN), a longitudinal cohort of 2,375 perimenopausal women (42–52 years old), showed that a rise in FSH over 4 years predicted a decline in BMD (27). The significant association between high serum FSH and bone resorption markers or BMD, particularly within the highest quartiles of serum FSH, was also noted in perimenopausal Chinese women (28, 29, 30, 31). The Bone Turnover Range of Normality (BONTURNO) study also showed that women with serum FSH levels >30 IU/L had higher bone turnover markers than age-matched women with FSH levels <30 IU/L (32). Likewise, in NHANES III, high serum FSH was significantly correlated with high bone turnover markers and low BMD (33), independent of estrogen. Similarly, our own study using the AGES–Reykjavik cohort of older adults from Iceland showed an inverse correlation between serum FSH and BMD and between serum FSH and incident hip fracture risk in elderly women, yet again independent of estrogen (34).

Furthermore, amenorrhea-related alterations in BMD were investigated in a study that reported greater bone loss in hypergonadotropic (elevated FSH) amenorrheic women compared to hypogonadotropic amenorrheic women. Elevated FSH levels were negatively correlated with bone density (35). In Turner syndrome, characterized by hypergonadotropic hypogonadism, patients with high levels of serum FSH showed elevated levels of the bone resorption marker, C-telopeptide, and increased osteoclastogenesis based on cultures of peripheral blood mononuclear cells (PBMCs). However, there was no correlation between 17β-estradiol levels and the number of osteoclasts (36). An observational study has further shown an inverse correlation between FSH levels and BMD *Z*-score in postpubertal Turner syndrome patients (37).

In an interventional study, postmenopausal women were treated with a GnRH agonist, leuprolide acetate, or placebo injections, with both groups receiving the aromatase inhibitor, letrozole, to eliminate variations in endogenous estrogen levels as a confounder (38). In the GnRH group, while suppression of FSH secretion did not reduce the levels of resorption markers, serum PINP, a bone formation marker, was increased significantly by ∼15% from baseline. The multiplicity of actions of the GnRH agonist on other hormones, such as LH, or the action of GnRH itself, might account for the unexpected action on resorption. But it is clear that lowering serum FSH does increase PINP, a validated bone formation marker. This is in line with bone-forming actions of FSH inhibition in rodent models.

In addition, it has been reported that genetic variants in the *FSHR* gene contribute to changes in bone turnover markers and BMD. The missense rs6166 polymorphism (Ser680Asn) in the coding region of the *FSHR* gene is associated with low BMD and high bone turnover in postmenopausal women. Namely, individuals with the AA genotype exhibited significantly lower BMD, elevated bone turnover markers, and increased risk of osteoporosis compared to those with the GG genotype. These associations were independent of circulating FSH and estrogen levels, suggesting a direct role of the rs6166 polymorphism in bone mass regulation in humans (39). In addition, digenic combinations between wild-type genotype of the 3′UTR and *IVS4* markers for the *CYP19A1* (aromatase) gene, and the *BMP15* and *FSHR* genes have been described as being osteoprotective (40).

Supporting the epidemiologic and genetic evidence for a role of FSH in bone mass regulation in humans are compelling pre-clinical mechanistic studies. As noted above, FSHRs are present on osteoclasts and osteoblast precursors, but are absent from mature osteoblasts in both humans and mice (19, 41). FSH binding to bone tissue has been documented *in vivo* using a near-infrared fluorophore conjugate of FSH (42). Specifically, osteoclasts and their precursors express Gi2α-coupled FSHRs that activate downstream MEK/ERK, NF-κB, and AKT signals, thereby promoting osteoclastogenesis and bone resorption (41). Haploinsufficient *Fshb*^+/−^ mice with intact ovarian function exhibit increased bone mass and reduced osteoclastic bone resorption (41). Elevated FSH levels promote the differentiation of osteoclast precursors by increasing RANK expression on CD14^+^ monocytes (20). Besides its direct action on osteoclasts, FSH also indirectly promotes osteoclast formation by stimulating the release of osteoclastogenic inflammatory cytokines. It triggers pre-osteoclasts to secrete IL-1β, TNF-α, and IL-6, which, in turn, correlate with the level of FSHR expression in monocytes (20, 43). The pro-osteoclastogenic response to FSH was not seen in mice lacking immunoreceptor tyrosine-based activation motif (ITAM) adapter signaling molecules (44), suggesting an interaction with immune receptors. Finally, FSHRs were found to be expressed on human osteoblast precursors and on stromal cells isolated from mice (45). Blocking FSH by anti-FSH antibody increased the number of osteoblast precursors, upregulated osteoblastogenic gene expression, and promoted bone formation (19, 45, 46).

Due to the reciprocal relationship between gonadotropins and sex hormones, it has been challenging to distinguish their independent effects on the skeleton. Accordingly, prior studies have reported conflicting findings. For example, a gain-of-function model using transgenic female mice expressing human FSH (TgFSH) and direct FSH administration in male mice produced distinct skeletal phenotypes. In two TgFSH mouse models with moderate or high FSH expression, the increased ovarian function was associated with dose-dependent increases in bone mass (47). However, the direct administration of FSH resulting in similar increase in serum FSH levels did not show a significant increase in bone mass (48). In one study, *Fshr*^−/−^ or *Fshb*^−/−^ mice with chronic estrogen deficiency displayed bone loss with aging. Ovarian transplantation in *Fshr*^−/−^ mice increased bone mass, suggesting a dominant effect of estrogen (49). Overall, with ovaries intact and producing estrogen in response to FSH, or when severe FSH deficiency leads to a compensatory increase in androgens, it is difficult if not impossible to isolate a direct action of FSH on the skeleton (50).

That said, three lines of evidence provide compelling evidence for a direct action of FSH on the skeleton. First, mice haploinsufficient in *Fshb*, or *Fshb*^+/−^ mice, in which sex steroids are unaltered with a ∼50% reduction in FSH, show a clear increase in bone mass, separating the effects of estrogen from those of FSH. Second, rodents receiving 4-vinylcyclohexene diepoxide (VCD), an ovatoxin, to mimic the perimenopausal transition where estrogen is preserved while FSH is elevated show clear evidence for bone loss (51). Finally, as noted below, we have selectively targeted FSH using an FSH-blocking antibody, which prevents bone loss arising from ovariectomy.

## Negative metabolic effects of FSH

The SWAN study showed that elevated FSH levels are positively correlated with waist circumference and fat mass during the perimenopausal transition, while estrogen levels remain stable (52, 53). Serum FSH is shown to be a better predictor of high fat mass than estrogen. The AGES–Reykjavik study showed that elderly women in the highest FSH quartile had higher bone marrow adiposity (34), and a study using a Chinese cohort reported that subjects with high FSH had high BMIs (54). This observation also holds true in the male population. A large observational study from China showed a positive correlation between FSH levels and BMI in older men (54). Importantly, in an interventional study, patients who underwent surgical orchiectomy for prostate cancer gained significantly more weight and subcutaneous and visceral fat mass compared with patients who received a GnRH agonist, suggesting that high FSH post-orchiectomy is associated with a body composition alteration in hypogonadal patients (55). It also suggests that suppressing FSH, when the adiposity is FSH-driven, would prevent fat mass accrual.

As discussed earlier, the FSHR is also expressed in adipocytes in different species, namely mice, boar, and humans (16, 54, 56, 57, 58). Similar to osteoclasts, the adipocyte FSHR is coupled to Giα, and its activation downregulates cAMP production (16). Thus, cAMP-mediated β3-adrenergic receptor signaling is decreased, which results in the downregulation of UCP1 (16, 59). On the other hand, FSH induces lipid synthesis by upregulating peroxisome proliferator-activated receptor gamma (PPARγ), CCAAT enhancer binding proteins (C/EBPα), lipoprotein lipase (LPL), and fatty acid synthase (FAS), increasing lipogenesis (16, 54). Blocking FSH action genetically in *Fshr*^+/−^ mice or pharmacologically using anti-FSH antibodies in high-fat-diet-treated or ovariectomized mice, stimulates UCP1 expression and mitochondrial biogenesis, resulting in increased energy expenditure and reduced fat mass (16, 41, 45). These findings suggest that *in vivo* FSH blockade may represent a therapeutic approach to both increase bone mass and reduce body fat. Notably, FSH inhibition did not suppress appetite; mice rather tended to eat more yet lost weight (16).

## FSH triggers cognitive decline

Alzheimer’s disease (AD) disproportionately affects women in terms of lifetime risk, rate of progression, and symptom burden, suggesting that the female sex could be a major risk factor for developing AD, particularly after menopause (60, 61, 62). It has been widely believed that estrogen deficiency underlies the preponderance of AD in postmenopausal women. However, studies examining the relationship between postmenopausal cognitive impairment and hormone replacement therapy have yielded conflicting outcomes, ranging from improvement (63, 64) to no change (65, 66) to even a decline in memory function (67, 68). This uncertainty regarding a role for estrogen has prompted a closer examination of other hormones, such as FSH, as a potential driver of cognitive decline in postmenopausal women (69, 70).

The SWAN and Penn Ovarian Aging Study provide the most compelling evidence for an association between rising FSH levels and cognitive decline (71, 72). The SWAN documented that women display mild cognitive impairment during late perimenopause, when FSH is rising in the face of normal estrogen levels (26, 73, 74). In addition, FSH is elevated in the cerebrospinal fluid of postmenopausal women, and correlates with increases in brain amyloid, reduced gray matter, and impaired cognition (75). Moreover, in women, two non-synonymous SNPs (rs6165 and rs6166) corresponding to substitutions at codon 307 (T/A) and codon 680 (S/N) in the *FSHR* gene (i.e. *FSHR* A307/S680 variant) have been associated with a lower AD risk (76).

FSHR expression was found in AD-vulnerable regions of mouse and human brains: these regions, namely the entorhinal cortex and the granular layer of the dentate gyrus of the hippocampus, are involved in learning behaviors and memory (77, 78). In a study using AD-prone *3xTg* transgenic mice, we found that elevated FSH levels following ovariectomy or intraperitoneal FSH injections caused a rapid accumulation of Tau and Aβ with impaired spatial learning and memory retrieval (15). Consistent with the hypothesis that FSH promoted AD-like pathology and memory loss, the downregulation of *Fshr* in the hippocampus or injection of our FSH-blocking antibody attenuated the memory loss and neuropathology in ovariectomized *3xTg* mice (15). Importantly, we found that *Fshr* deletion on a *3xTg* background resulted in a gene-dose-dependent rescue of spatial and recognition memory in *3xTg*;*Fshr*^+/−^ and *3xTg*;*Fshr*^−/−^ mice compared with *3xTg*;*Fshr*^+/+^ mice (79). FSH administration also exacerbated the AD-like pathology in *ApoE4* knock-in mice, suggesting that FSH might have an additive effect on the known propensity of the *ApoE4* phenotype in AD pathogenesis (80).

## Extragonadal expression and functions of LHCGR

The LHCGR has also been shown to be expressed in a variety of extragonadal tissues (81). In the human female reproductive system and placenta, LHCGR expression has been identified in endometrial epithelial and stromal cells, cervix, myometrium, and trophoblast cells, indicating that LH may also exert biological effects in extragonadal reproductive tissues (82, 83). In the placenta, LHCGR signaling is tightly controlled by a feedback mechanism that limits receptor responsiveness after activation, which seems to be lost in pathological trophoblast-derived choriocarcinoma cells (84, 85). The LHCGR is also expressed in the adrenal cortex, particularly in the zona reticularis, and is thought to be linked to the production of dehydroepiandrosterone sulfate (DHEAS) (86).

In addition, we also found LHCGR expression in multiple regions of the central nervous system, namely, the hippocampus, hypothalamus, and cerebral cortex (78). Given that these brain regions are critically involved in cognitive function, sleep–wake regulation, and behavioral control, the LH–LHCGR axis may influence cognitive processes and sleep regulation. In support of this notion, experimental modulation of gonadotropin signaling has been shown to alter sleep–wake patterns and hippocampus-associated behaviors (87, 88). Moreover, alteration in LH levels and LH–LHCGR signaling have been associated with neurodegenerative processes, particularly Alzheimer’s disease and dementia (89, 90).

Evidence for LHCGR expression and action in skeleton and fat is currently limited. LHCGR is expressed in human adipose tissue, with expression that is higher in mature adipocytes than in precursor preadipocytes (91). hCG injections were used for weight loss without a clear scientific basis (92, 93). In a small *in vitro* study, hCG was shown to promote preadipocyte growth and differentiation through the activation of the MEK–MAPK/ERK pathway (91). There are data on LHCGR expression in bone that have been detected both at the RNA and protein levels, but its functional significance remains unclear (94). Genetically modified mice with up- or downregulated LH signaling pathways, namely hCG overexpressing transgenic mice and LHRKO mice, showed striking skeletal phenotypes. LHRKO mice had reduced BMD and lower histomorphometric indices in male and female mice, whereas hCG transgenic mice showed increased BMD, particularly in females (95). However, these findings should be interpreted with caution, as significant alterations in testosterone and estrogen levels are obvious confounders. Further studies are needed to better define the direct role of LHCGR signaling in skeletal remodeling and adipogenesis.

## Targeting FSH as a potential therapeutic for osteoporosis, obesity, and dementia

Based on pre-clinical and human studies, FSH has become an actionable target for osteoporosis, obesity, and AD. Our original polyclonal and monoclonal antibodies target a computationally defined 13-mer epitope (LVYKDPARPKIQK) within the receptor-binding domain of human FSHβ (16, 17, 22, 45). These antibodies increase bone mass in wild type and ovariectomized mice through both anti-resorptive and anabolic actions (17, 22, 46). In addition, blocking FSH action prevented fat gain and induced beiging in all fat depots in mice on a high-fat diet or post-ovariectomy (16). In studies from other labs, vaccination with tandem repeats of the same epitope in mice prevented fat accrual and induced beiging (96). Boars vaccinated with these tandem repeats gained less fat compared with castrated pigs where FSH levels are high (58). Blocking FSH action also prevented the onset of AD-like neuropathology – Aβ plaque and neurofibrillary tangle formation – and memory loss in the ovariectomized 3x*Tg* mouse (15), while simultaneously reducing neuronal apoptosis and increasing dendritic spine and synapse number, with a clear effect in preventing spatial memory loss in the Morris Water Maze test (15).

Our lead humanized candidate, Hu6, was chosen from a panel of 30 humanized FSH antibody clones as one that displayed the highest binding affinity (K_D_) of 7.53 nM (46, 97). Using HEK cells overexpressing the FSHR, we found that Hu6 directly prevented FSH binding to the FSHR, and in doing so, attenuated osteoclastogenesis in a bone marrow cell culture assay and inhibited the expression of beiging genes *in vitro* in 3L3 adipocytes (46). We also found that Hu6 stimulated bone formation in wild-type Thermo mice, as well as in C57BL/6 mice that have been ovariectomized and allowed to lose bone, consistent with an anabolic action (98). We have also developed a clinical-grade formulation and performed a range of biophysical tests for antibody stability. We find that Hu6 displays thermal, monomeric, colloidal, structural, and accelerated stability, as well as acceptable levels of viscosity, clarity, and turbidity at an ultra-high concentration of up to 150 mg/mL in formulation, which is suitable for human use (99, 100). Finally, studies in African green monkeys have shown no evidence of deleterious effects on vital signs, serum chemistries, or blood counts (97, 98).

## Conclusion

**Figure 1:**
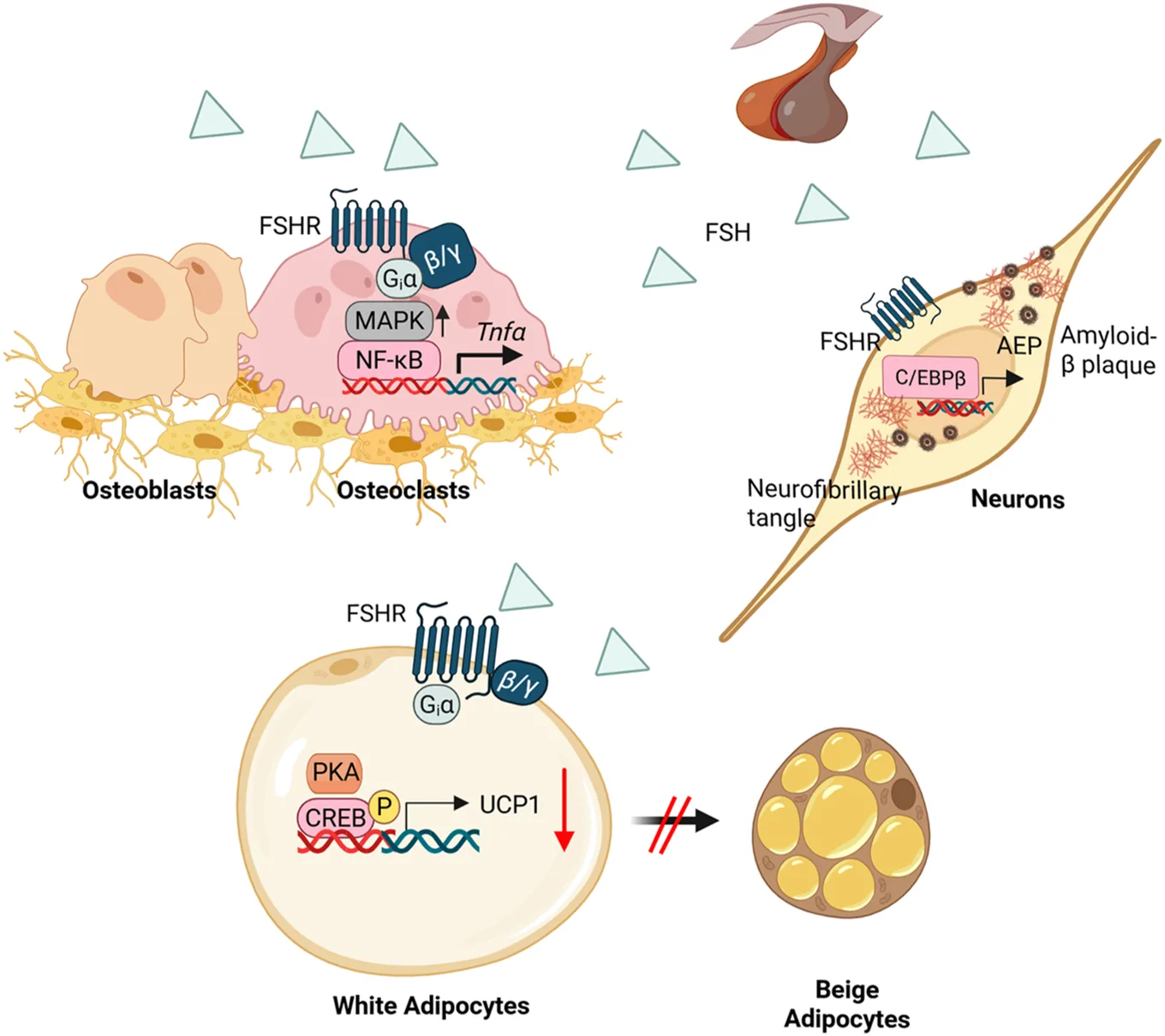
Non-gonadotropic effect of FSH. FSH receptors (FSHRs) on osteoclasts induce TNFα expression through NF-κB, resulting in increased osteoclast differentiation. In adipocytes, FSH action inhibits beiging and thermogenesis. FSHRs in osteoclasts and adipocytes are coupled with inhibitory Gi, unlike in ovaries, where they are coupled with stimulatory Gs. In neurons, FSH activates C/EBPβ-induced asparagine endopeptidase (AEP), increasing neurofibrillary tangles and amyloid β plaques. Mitogen-activated protein kinase (MAPK); CCAAT/enhancer-binding protein beta (C/EBPβ); protein kinase A (PKA); cAMP response element-binding protein (CREB); uncoupling protein 1 (UCP1).

The gonadotropins, FSH and LH, traditionally regarded as regulators of reproductive function, are now increasingly recognized to exert direct effects in extragonadal tissues, including bone, adipose tissue, and the central nervous system. Epidemiologic, genetic, and experimental evidence suggests that elevated FSH contributes to skeletal loss, increased adiposity, and neurodegenerative changes during the perimenopausal transition, independent of declining estrogen (Fig. 1). Leveraging pre-clinical mechanistic studies, blocking FSH action by a humanized antibody has gained traction as a potential therapeutic. Although the functional significance of extragonadal LHCGR signaling remains less well defined, emerging data suggest possible roles in skeletal and adipose remodeling that warrant further investigation. Together, these findings challenge the traditional paradigm that gonadotropins act solely through gonadal steroidogenesis and support the concept that targeting gonadotropin signaling, particularly FSH, may offer a novel therapeutic approach for age-associated disorders affecting bone, metabolic, and neurocognitive health. A question, thus, arises: should we consider renaming gonadotropins to reflect their broader biological functions.

## Declaration of interest

M Z is an inventor on issued patents on inhibiting FSH for the prevention and treatment of osteoporosis and obesity (US Patent Nos. 8,435,948 and 11,034,761). M Z is also an inventor on a pending patent application on the composition and use of humanized monoclonal anti-FSH antibodies and is a co-inventor of a pending patent on the use of FSH as a target for preventing Alzheimer’s disease. These patents are owned by Icahn School of Medicine at Mount Sinai (ISMMS), and M Z would be recipient of royalties, *per* institutional policy. M Z also consults for several financial platforms, including Gerson Lehrman Group and Guidepoint, on drugs for osteoporosis and genetic bone diseases.

## Funding

Work at Icahn School of Medicine at Mount Sinai performed at the Center for Translational Medicine and Pharmacology was supported by R01 AG071870 to M Z, T Y, and S-M K; R01 AG074092 and U01 AG073148 to T Y and M Z; U19 AG060917 to M Z. M Z also thanks the Harrington Discovery Institute for the Innovator–Scholar Award.
